# Supplementary material for: Risk Prediction for the Development of Hyperuricemia: Model Development Using an Occupational Health Examination Dataset
Source: Int J Environ Res Public Health. 2023 Feb 15;20(4):3411. doi: 10.3390/ijerph20043411 (PMC9967697; doi:10.3390/ijerph20043411)
Supplement: Supplementary file 1 [file ijerph-20-03411-s001.zip › Supplementary Table S1 Variable Filter Forecast Table.pdf]

Supplementary Table S1. Variable Filter Forecast Table

Variable	Value
Cr（U/L）	10.278
BMI（kg/m^2）	7.863
DASH	-4.204
Waistline(cm)	2.221
Diastolic pressure（mmHg）	2.013
TC（mmol/L）	0.638
